# Prevalence and antimicrobial susceptibility patterns of *Salmonella* and *Shigella* isolates among children aged below five years with diarrhea attending Robe General Hospital and Goba Referral Hospital, South East Ethiopia

**DOI:** 10.1186/s40794-019-0096-6

**Published:** 2019-11-20

**Authors:** Addisu Assefa, Mengistu Girma

**Affiliations:** 1 Department of Biology, Madda Walabu University, P.O.B.247, Bale Robe, Ethiopia; 2 Robe Teachers College, Bale Robe, Ethiopia

**Keywords:** Children, Drug susceptibility test, Prevalence, Associated factors, *Salmonella*, *Shigella*, Ethiopia

## Abstract

**Background:**

Diarrheal diseases are responsible for high level of morbidity and mortality, particularly in children below 5 years. *Salmonella* and *Shigella* spp. are pathogenic microbes responsible for the major diarrheal associated mortality. The purpose of this study was to determine the prevalence, factors associated with *Salmonella* and *Shigella* isolates infections and their antimicrobial susceptibility patterns among diarrheic children aged below 5 years attending BRGH and GRH, Ethiopia.

**Methods:**

A health institution based cross-sectional study was conducted from April to July 2016. One stool samples was collected from 422 diarrheic children under the ages of five and were cultured on to Hektoen Enteric (HE) and Salmonella-Shigella agar. Isolation identification of the *Salmonella* and *Shigella* isolates were conducted using standard bacteriological methods. Antibiotic susceptibility was done by Kirby–Bauer disk diffusion method. The isolates were defined as multidrug resistant if it was resistant to two or more antimicrobial agents. Descriptive statistics were employed and logistic regression models were constructed to determine factors associated with *Shigella/Salmonella* prevalence.

**Results:**

The prevalence of *Salmonella* and *Shigella* isolates were 6.9 and 4.3%, respectively. Children aged between 1 to 3 years were significantly associated with *Salmonella* infection [AOR = 19.08, 95% CI (2.68–135.86)]. The odd of prevalence of *Salmonella/Shigella* isolates was significantly associated with absence of latrine, absence of hand washing after latrine, and in unimmunized children in adjusted odd ratio. Unimproved water sources and hand washing before meal had also higher odd of prevalence although the difference was not significant. All *Salmonella* and *Shigella* isolates were resistant to amoxicillin (100%). In addition, all *Shigella* isolates were completely resistant to chloramphenicol, and tetracycline, and were multidrug resistant. However, all *Salmonella* and *Shigell*a isolates were susceptible to ciprofloxacin and ceftriaxone.

**Conclusion:**

There was a relatively low prevalence of *Salmonella* and *Shigella* species in the study areas and were significantly associated with lack of personal hygiene and environmental sanitation. There were also higher drug resistance and multidrug resistant pattern. Personal hygiene and environmental sanitation, including access to latrine and supply of safe drinking water are suggested. Checking susceptibilities of *Shigella* and *Salmonella* isolates causing diarrhea is also suggested.

## Background

Diarrhea is a major cause of morbidity and mortality among children < 5 years old in sub-Saharan Africa [1]. It is the second cause of death (16%) after pneumonia in children under 5 years of age worldwide, with developing countries being the most affected [2]. The most common causes of infectious diarrhea include: viruses, bacteria, protozoa and unidentified and mixed infections [3]. Among bacterial pathogens, *Salmonella* and *Shigella* are of particular concern as causes of enteric fevers, food poisoning and gastroenteritis [3, 4].

*Salmonella*, with its more than 2500 different serotypes, is a leading cause of food-borne infections worldwide. *Salmonella* spp. causes self-limiting gastro-enteritis and the more severe forms of systemic typhoid fever. Typhoid fever is principally caused by *Salmonella enteric* serotype Typhi in humans. *Salmonella* outbreaks are related to unhygienic food preparation, cooking, reheating and storage practices that are contaminated with the pathogen [3, 4]. In addition, poor access to good latrine, poor sanitation and hygienic status, hand washing habit before and after meal and / or latrine, absence of proper sewage disposal system were responsible for typhodial type of salmonella infections [5–9]. Shigellosis is caused by *Shigella* spp. and it is a worldwide problem although more prevalent in developing countries [10–12]. Shigellosis is common in areas where living standards are very low and access to safe and adequate drinking water and proper waste disposal systems are often very limited, or even absent [6–9, 13–17]. *Shigella* spp. are limited to the intestinal tract of humans and cause bacillary dysentery leading to watery or bloody diarrhea.

Studies conducted in Ethiopia also revealed an increasing trend in the prevalence of *Salmonella* isolates and *Shigella* spp. [5–9, 14]. Antimicrobial resistance of *Salmonella* and *Shigella* are emerging global challenges, especially in developing countries where there is an increased misuse of antimicrobial agents in humans and animals [10, 11]. In Ethiopia in the past three decades, studies indicated that *Salmonella* and *Shigella* have developed varied rate of resistance against the first line antibiotics such as ampicillin, tetracycline, co-trimoxazole, chloramphenicol [5–9, 13, 14, 16–22], second generation fluoroquinolones such as norfloxacin and/or ciprofloxacin [7, 9, 13, 16, 19, 20, 22], the third generation cephalosporins (ceftriaxone) [7–9, 18, 20]. However, there is no published information on the magnitude of *Salmonella* and *Shigella* infection and their response to commonly prescribed drugs among diarrheic children aged below 5 years in Bale Robe General Hospital (BRGH) and Goba Referral Hospital (GRH), South Eastern Ethiopia. Understanding the magnitude and drug susceptibility pattern of *Salmonella* and *Shigella* are important in designing public health intervention measure in the area. The purpose of this study was to therefore determine the prevalence, associated factors and antimicrobial susceptibility patterns of *Salmonella* and *Shigella* isolates among children aged below 5 years with diarrhea in RGH and GRH, South Eastern Ethiopia from April 2016 up to July 2016.

## Methods

### Study area, design and period

Institution based cross-sectional study was conducted at BRGH and GRH from April 2016 up to July 2016 in order to determine the prevalence and antimicrobial susceptibility patterns of *Salmonella* and *Shigella* species in diarrheic children under the ages of five. BRGH and GRH are located are 430 Km and 445 Km, respectively away from Addis Ababa in South Eastern Ethiopia. The two hospitals provide health services for two major towns of the zones and to the surrounding districts.

### Study population

The source population was all children under the ages of five and who lived either in Robe town, Goba town or in the adjacent rural villages during the study period. The study population was all diarrheic children under the ages of five in the selected hospitals who obtained medical service during the study period. Study population who had taken antibiotics 2 weeks before the study and with incomplete demographic information were excluded from the study.

### Sample size determination and sampling techniques

The sample size (n) was determined though a single proportion formula by taking an estimated prevalence of 0.5 (for unknown prevalence) [23]. In addition, 10% of the sample size was added to the normal sample size to minimize errors [24]. A total of 422 study population was included in the study both for socio-demographic survey and stool sample collection. Simple random sampling methods were used to sample study population. Accordingly, 283 and 139 study participants were enrolled from Goba Referral Hospital and Robe General Hospital in the study, respectively.

### Data and specimen collection

A structured questionnaire was used to collect data on socio-demographic characteristics (age, sex and residence) of the study population (children), and associated factors (status of drinking water, availability of latrine, hand washing habit before meal and after toilet, immunization status, availability of waste disposal system, presence of domestic animal and milk treatment status). Such data were retrieved from children parents or guardians by experienced physician or nurse (Table 1; Additional file 1). These factors were selected based on literature survey and considering personal hygiene habits and environmental sanitation of the people in the study areas. Children parents or guardians were also informed to bring freshly passed stool and rectal swab of the study subjects in a sterile stool cup using clean applicator stick. Accordingly, one stool sample was collected from 422 children under the age of five. The collected stool was placed immediately in Cary Blair transport (Oxoid, UK) and transported to laboratory for immediate processing. Instructions were given to children parents or guardians on how to collect stool samples from study children.

**Table 1:** Socio-demographic characteristics and prevalence of *Salmonella* and *Shigella* isolates

Variable	Patient Frequency	Percentage	*Salmonella* isolates Positive isolates (%)	*Shigella* isolates Positive isolates (%)
Hospital				
Goba	283	67.1	22 (7.8)	15 (5.3)
Robe	139	32.9	7 (5.0)	3 (2.1)
Sex				
Male	179	42.4	14 (7.8)	7 (3.9)
Female	243	57.6	15 (6.2)	11 (4.5)
Residence				
Urban	176	41.7	6 (3.4)	4 (2.3)
Rural	246	58.3	23 (9.3)	14 (5.7)
Age				
< 1 year	129	30.6	2 (1.6)	0 (0)
1–3 year	86	20.4	11 (12.8)	6 (7.0)
3–5 Year	207	49	16 (7.7)	12 (5.8)
Potable water^a^				
Improved	343	81.28	16 (4.7)	7 (2.0)
Unimproved	79	18.72	13 (16.5)	11 (13.9)
Availability of latrine				
Yes	250	59.24	7 (2.8)	3 (1.2)
No	172	40.75	14 (8.1)	15 (8.7)
Hand wash before meal				
Yes	381	90.3	22 (5.8)	12 (3.1)
No	41	9.7	7 (17.1)	6 (14.6)
Hand wash after latrine				
Yes	342	81.04	10 (2.9)	8 (2.3)
No	80	18.96	19 (23.8)	10 (12.6)
Immunization				
Yes	311	73.7	12 (3.9)	6 (1.9)
No	111	26.3	17 (15.3)	12 (10.8)
Availability of waste disposal				
No	183	43.36	20 (10.9)	15 (8.2)
Yes	239	56.64	9 (3.8)	3 (1.3)
Domestic animal presence				
Yes	166	39.34	13 (7.8)	10 (6.0)
No	256	60.66	16 (6.2)	8 (3.1)
Milk status				
Boiled	146	34.6	5 (3.4)	1 (0.7)
Unboiled	276	65.4	24 (8.7)	17 (6.2)
Total	422	100	29 (6.9)	18 (4.3)

Abbreviations: ^a^refers to unimproved water (drank raw without any chemical treatment) or improved (water drank after chemical treatment)

### Culture and identification

Approximately, 1 g of stool specimen was suspended in Selenite-Cystine broth (Oxoid, UK) contained in sterile test tube for overnight. A loopful of the suspension of the specimen was streaked on to two different media namely, Hektoen Enteric (HE) Agar and Salmonella-Shigella (SS) agar, both from Oxoid, UK. These plates were incubated aerobically at 37 °C overnight to enhance the recovery of the two pathogens. The isolates were purified by subculturing on nutrient agar plates. A colorless colony with or without black center on SS agar media, and a blue green colony with or without black center on HE agar were presumably isolated as Salmonella-like isolates. A colorless colony on SS and a green, moist and raised colony on HE agar were presumably isolated as Shigella-like isolates [3, 25, 26]. Colonies exhibiting characteristic reactions of Salmonella and Shigella-like were further characterized by the pattern of biochemical reactions after inoculation on to Triple sugar iron agar, lysine iron agar, Simon’s citrate agar, and MIU test (motility test, Indole and Urease production) for final identification using the standard procedures [3, 25, 26].

### Antibiotic susceptibility pattern

The antimicrobial susceptibility testing of *Salmonella* and *Shigella* isolates was done by Kirby- Bauer disc diffusion method according to Clinical and Laboratory Standards Institute [27] guidelines. Colonies from pure cultures of *Salmonella* or *Shigella* were taken and transferred to a tube containing 5 ml sterile distilled water. It is mixed gently until a homogenous suspension was formed. The suspension was incubated at 37 °C until the turbidity became adjusted to a 0.5 McFarland standard and then was uniformly inoculated on to Muller Hinton Agar (Oxoid, UK) by using sterile cotton swab under laminar hood. The inoculated plates were left at room temperature to dry for 3–5 min A sterilized forceps was then used to lightly press the antibiotic discs manually on the surface of a Muller-Hinton plate to make firm attachment. Accordingly, each isolate was subjected to 11 antibiotics discs on Muller Hinton agar (Oxoid, UK): amoxicillin (AML) (2 μg), ceftriaxone (CTR) (30 μg), ciprofloxacin (CIP) (5 μg), chloramphenicol (C) (30 μg), doxycycline (DO) (30 μg), and tetracycline (TE) (10 μg) (all from Oxoid, UK). The plates were then incubated at 37 °C for 24 h. The control disc was impregnated with sterile distilled water. Diameters of the zone of inhibition was measured to the nearest millimeter using a metallic caliper, and the isolates were classified as sensitive, intermediate and resistant according to the standardized table supplied by the CLSI [27]. *Salmonella*/*Shigella* isolate was defined as multidrug resistant if it was resistant to two or more antimicrobial agents tested [9, 18, 28].

### Data quality control

In order to generate quality and reliable data, all questions were prepared in a clear and precise way and translated into two local languages (Afan Oromo and Amharic). Completeness of the questionnaire was checked whether the necessary information was properly full filled or not. All the instruments used for sample processing were checked for sterility and proper functioning. For quality control, strains of *E. coli* ATCC 25922, *Shigella flexneri* ATCC 12021, and *Salmonella* Typhimurium ATCC 14028 were obtained from Ethiopian Public Health Institute. The sterility of prepared media was checked by incubating one of the prepared media for 24 h at 37 °C. Growth media that showed bacterial growth were discarded.

### Data analysis

The data was analyzed using IBM SPSS Statistics for Windows version 20 (IBM Corp., Armonk, NY, USA). Prevalence/ isolation rate (the outcome or dependent variable) was expressed as percentage of stool samples of study subjects showing cultured-confirmed *Salmonell*a or *Shigella* isolates divided by the total number of screened stool samples of study subjects. Socio-demographic factors and associated factors were independent factors from which predictors factors for the outcome variables were identified. Descriptive statistics was used to analyze the rate of isolation of *Salmonell*a or *Shigella* isolates. Bivariate and multivariable logistic regression analyses were used to compute crude ratio (COR) and adjusted odds ratio (AOR). The independent variables were checked for the presence of little or no multicollinearity using Variance Inflation Factor (VIF) (VIF = 1, not correlated; between 1.0–5.0, moderately correlated and > 5.0 highly correlated). The goodness of fit of the employed model was evaluated using the Hosmer-Lemeshow test. A *p*-value > 0.05 indicated that the model fit well to run the logistic regression analysis. The logistic regression analysis was done by step-wise manner. First, each independent variable and outcome variable was first evaluated by bivariate logistic regression to calculate Crude odd ratio (COR). The COR of independent variables whose 95% confidence interval (CI) excluding one were fitted to multivariable logistic regression to calculate the adjusted odd ratio (AOR). In AOR, the 95% confidence interval (CI) of odds ratio (OR) excluding one was significantly associated with the corresponding dependent variable.

### Ethical consideration

This study was approved by College of Natural and Computational Science of Madda Walabu University. Ethical clearance was obtained from Bale Zone Health Office. Permission to conduct this study was obtained from each hospital administration. Both oral assent and written informed consent were obtained from the parents or guardians of the study subjects before administration of the study. Information of the study subjects and that of the parents or guardians of the study subjects were kept confidential.

## Results

### Socio-demographic data

A total of 422 stool samples were collected from study participants in BRGH (*n* = 139) and GRH (*n* = 283) and the response rate was 100%. Among study subjects, 179 (42.4%) were male and 243 (57.6%) were female. Out of the total study subjects, 176 (41.7%) and 246 (58.3%) lived in urban and rural areas, respectively. The age of the study participants ranged from 3 months to 60 months (mean age 31 months ±4.7 months). Among them, 129 (30.6%) were infants, 86 (20.4%) were between 1 to 3 years and 207 (49%) were between 3 to 5 years. Most of the participants utilized an improved water (81.28%). Availability of latrine, handwashing before meal and after toilet, and child immunization were recorded from 59.24, 90.3, 81.04 and 73.7% of the respondents, respectively (Table 1).

### Prevalence of *Salmonella* and *Shigella* isolates

The isolation rate of *Salmonella* and *Shigella* isolates are shown in Table 1. *Salmonella* and *Shigella* isolates were positive in 29 (6.9%) and in 18 (4.3%) study subjects, respectively. 22 (7.8%) of the *Salmonella* isolates were from GRH, and 7 (5.0%) were from BRGH. Of the 18 *Shigella* isolates, 15 (5.3%) were from GRH and 3 (2.2%) were from RGH. A higher isolation rate of *Salmonella* and *Shigella* isolates were recorded from study subjects in rural areas (9.3 and 5.3%) than urban areas (3.4 and 2.3%) (Table 1).

### Associated factors for *Salmonella* and *Shigella* species infections

Associated factors for the prevalence of *Salmonella* and *Shigella* infection were shown in Tables 2 and 3, respectively. Children whose ages were between 1 to 3 years were 19.8 times [AOR = 19.8; 95% CI (2.68–135.86)] susceptible to *Salmonella* infection than children younger than 1 year. Absence of latrine and parents who didn’t wash their hands after latrine had 3.84 times [AOR = 3.84; 95% CI (1.31–11.2)], and 12.66 times [AOR = 12.66; 95% CI (4.54–35.3)], respectively higher odd of exposing their children to *Salmonella* infection. Children who drank from an unimproved water sources and parents who served foods to their children before washing their hands were more likely exposing their children to *Salmonella* infection although the difference was not significantly different from their counterparts. Absence of child immunization [AOR = 3.54; 95% CI (1.32–9.51)] was significantly associated with *Salmonella* infection) (Table 2).

**Table 2:** Prevalence of *Salmonella* isolates and associated factors using logistic regression

Variables	Patient Frequency	Positive isolates (%)	COR (95% CI Lower-Upper)	AOR (95% CI Lower-Upper)
Sex				
Male	179	14 (7.8)	1	
Female	243	15 (6.2)	0.77(0.36–1.65)	–
Age				
< 1 year	129	2 (1.6)	1	1
1–3 year	86	11 (12.8)	**9.31(2.01–43.16)**	**19.08 (2.68–135.86)**
3–5 Year	207	16 (7.7)	1.7 (0.77–3.94)	2.85 (1.0–8.09)
Residence				
Urban	176	6 (3.4)	1	1
Rural	246	23 (9.3)	**2.92 (1.16–7.33)**	0.43 (0.11–1.68)
Potable water^a^				
Improved	343	16 (4.7)	1	1
Unimproved	79	13 (16.5)	2.24 (0.86–5.82)	1.5 (0.48–4.69)
Latrine availability				
Yes	250	7 (2.8)	1	1
No	172	14 (8.1)	**5.09 (2.12–12.20)**	**3.84 (1.31–11.2**)
Hand wash before meal				
Yes	381	22 (5.8)	1	1
No	41	7 (17.1)	**10.34 (4.58–23.3)**	1.92 (0.272–3.13)
Hand wash after latrine				
Yes	342	10 (2.9)	1	1
No	80	19 (23.8)	**3.36 (1.33–8.43)**	**12.66 (4.54–35.3)**
Immunization				
Yes	311	12 (3.9)	1	1
No	111	17 (15.3)	**4.50 (2.07–9.77)**	**3.54 (1.32–9.51)**
Availability of waste disposal				
No	183	20 (10.9)	1	1
Yes	239	9 (3.8)	0.32 (0.14–0.71)	0.73 (0.26–2.06)
Domestic animal presence				
Yes	166	13 (7.8)	1	–
No	256	16 (6.2)	0.78 (0.36–1.67)	
Milk status				
Boiled	146	5 (3.4)	1	1
Non-boiled	276	24 (8.7)	**2.68 (1.003–7.19)**	0.33 (0.081–1.39)

^a^refers to unimproved water (drunk raw without any chemical treatment) or improved (water drank after chemical treatment); *AOR* Adjusted Odd Ratio, *COR* Crude odd ratio. Factors of independent variables with bolded COR/AOR and confidence intervals were were significantly associated the dependent variable (*p*-value ≤0.05)

**Table 3:** Prevalence of *Shigella* isolates and associated factors using logistic regression

Variables	Patient Frequency	Positive isolates (%)	COR (95% CI Lower-Upper)	AOR (95% CI Lower-Upper)	*P*-Value
Sex					
Male	179	7 (3.9)	1	–	–
Female	243	11 (4.5)	1.16 (0.44–3.06)	–	–
Age					
< 1 year	129	0 (0)	–	–	–
1–3 year	86	6 (7.0)	1	–	–
3–5 Year	207	12 (5.8)	1.22 (0.44–3.36)	–	–
Residence					
Urban	176	4 (2.3)	1	–	–
Rural	246	14 (5.7)	2.59 (0.84–8.02)	**–**	**–**
Potable water^a^					
Improved	375	7 (2.0)	1		
Unimproved	47	11 (13.9)	2.40 (0.75–7.61)	1.54 (0.40–5.85)	
Latrine availability					
Yes	250	3 (1.2)	1	1	
No	172	15 (8.7)	**7.86 (2.24–27.6)**	**4.58 (1.1–18.9)**	
Hand wash before meal					
Yes	381	12 (3.1)	1	1	
No	41	6 (14.6)	**5.27 (1.86–14.9)**	2.38 (0.60–9.32)	
Hand wash after latrine					
Yes	342	8 (2.3)	1	1	
No	80	10 (12.59)	**5.96 (2.27–15.65)**	**3.31 (1.02–10.90)**	
Immunization					
Yes	311	6 (1.9)	1	1	
No	111	12 (10.8)	**6.16 (2.25–16.85)**	**3.63 (1.04–12.65)**	
Availability of waste disposal					
No	183	15 (8.2)	1	1	
Yes	239	3 (1.3)	0.142 (0.04–0.50)	0.22 (0.044–1.00)	
Domestic animal presence					
Yes	166	10 (6.0)	1	1	
No	256	8 (3.1)	0.50(0.19–1.30)	1.11(0.28–4.32)	
Milk status					
Boiled	146	1 (0.7)	1	1	
Unboiled	276	17 (6.2)	**9.51 (1.25–72.25)**	6.93 (0.72–66.78)	

Abbreviations: ^a^refers to unimproved water (drank raw without any chemical treatment) or improved (water drank after chemical treatment); *AOR* Adjusted Odd Ratio, *COR* Crude odd ratio. Factors of independent variables with bolded COR/AOR were significantly associated the dependent variable (*p*-value ≤0.05)

The odd of prevalence of *Shigella* infection were significantly higher in the absence of latrine [AOR = 4.58; 95% CI (1.1–18.9)], in children whose parents didn’t wash their hand after latrine [AOR = 3.31; 95% CI (1.02–10.90)]. Children who drank from unimproved water sources and that was served by parents who didn’t wash their hands before meal were also more likely exposed to *Shigella* infection although the difference was not significantly varied from their counterparts. Unimmunized children had about 3.63 times higher infection risk than those who were immunized [AOR = 3.63; 95% CI (1.04–12.65)] (Table 3).

### Antimicrobial susceptibility test

**Fig. 1:**
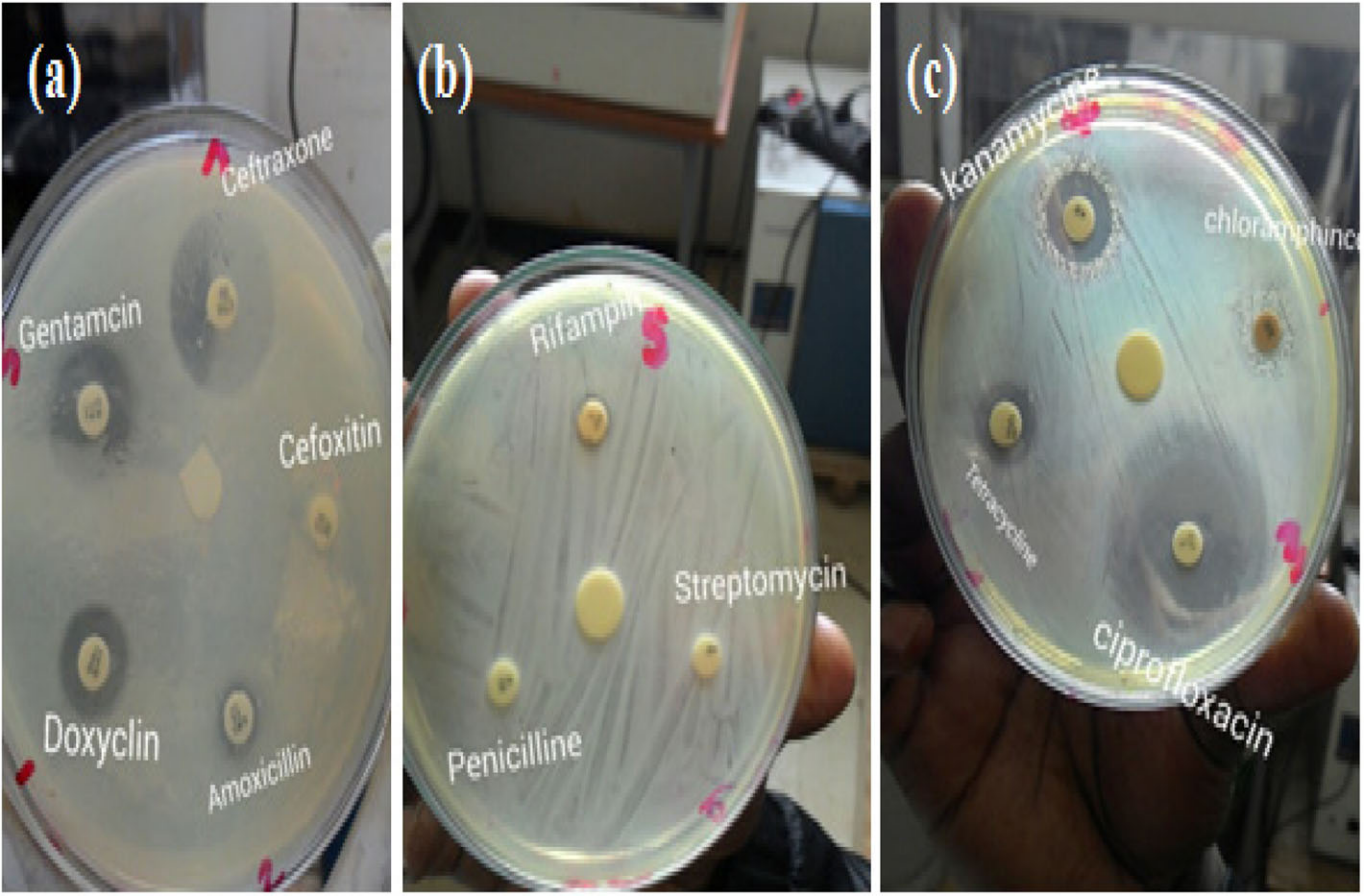
Antibiotic susceptibility pattern of *Shigella* isolates against 11 antimicrobial drugs using agar well diffusion method on three Petri Plates. Control discs are indicated as Control at the centre of the plate. They were impregnated with sterile distilled water

The *Salmonella* and *Shigella* isolates displayed different rate of susceptibility to the evaluted antibiotics (Fig. 1; Table 4). All *Salmonella* isolates (29) and *Shigella* isolates (18) were 100% susceptible to ciprofloxacin, and ceftriaxone (Table 4). However, *Salmonella* isolates showed high rate of resistance against amoxicillin (100%) and tetracycline (79.3%), while low resistance was observed against chloramphenicol (24.1%). *Salmonella* isolates showed 100% intermediate susceptibility against doxycycline. The *Shigella* isolates showed high rate of resistance against chloramphenicol, tetracycline, and amoxicillin (100% each) and to doxycycline (88.9%) (Table 4).

**Table 4:** Antimicrobial susceptibility pattern of *Salmonella* and *Shigella* isolates

Antimicrobials	*Salmonella* No. (%)			*Shigella* No. (%)		
	R	I	S	R	I	S
C	9 (24.1)	13 (44.8)	7 (24.15)	18 (100.0)	0 (0.0)	0 (0.0)
TE	23 (79.3)	6 (20.7)	0 (0.0)	18 (100.0)	0 (0.0)	0 (0.0)
DO	0 (0.0)	29 (100.0)	0 (0.0)	16 (88.9)	2 (11.1)	0 (0.0)
CIP	0 (0.0)	0 (0.0)	29 (100.0)	0 (0.0)	0 (0.0)	18 (100.0)
AML	29 (100.0)	0 (0.0)	0 (0.0)	18 (100.0)	0 (0.0)	0 (0.0)
CTR	0 (0.0)	0 (0.0)	29 (100.0)	0 (0.0)	0 (0.0)	18 (100.0)

*Abbreviations*: *R* Resistant, *I* Intermediate, *S* Sensitive, *C* Chloramphenicol, *TE* Tetracycline, *DO* Doxycycline, *CIP* Ciprofloxacin, *AML* Amoxicillin, *CTR* Ceftriaxone

The results of multiple drug resistant (MDR) patterns of *Salmonella* and *Shigella* are presented in Table 5. Most of the *Salmonella* isolates (*n* = 23, 79.31%) and *Shigella* isolates (*n* = 18,100%) were found to be multiple drug resistant (resistant to two and above antimicrobial drugs). 14 (48.3%) and 9 (31.03%) of *Salmonella* isolates displayed MDR against two and three drugs, respectively. On the other hand, 2 (11.1%) and 16 (88.9%) *Shigella* isolates were resistant to three and four drugs, respectively (Table 5).

**Table 5:** Multidrug resistant pattern of *Salmonella* and *Shigella* isolates

Number of antibiotics resisted	MDR Pattern	*Salmonella* isolates N (%)	*Shigella* isolates N (%)
2	AML, TE	14 (48.3)	
3	AML, C, TE	9 (31.03) ()	2 (11.1)
4	AML, C, TE, DO		16 (88.9)
Total		23 (79.31)	18 (100)

*Abbreviations*: *MDR* multidrug resistance, *N* Number of isolates resisted, *C* Chloramphenicol, *TE* Tetracycline, *DO* Doxycycline, *AML* Amoxicillin

## Discussion

This study was the first report on the prevalence and antimicrobial susceptibility pattern of *Salmonella* and *Shigella* isolates in RGH and GRH. The isolation rate of *Salmonella* isolates (6.9%) in our study (Table 1) was comparable with the study in Jimma, South west Ethiopia (6.2%) [6], but higher than Hawassa town, South Ethiopia (2.5%) [7], Kenya (3.5%) [29] and Botswana (3.0%) [30], and was lower than Bahir Dar, North Ethiopia (7.8%) [31] and Arba Minch, South Ethiopia (12.6%) [9]. *Shigella* isolates had the isolation rate of 4.3% (Table 1) in conformity with the results from Arba Minch (4.8%) [9] and Nepal (4.6%) [32], but it was lower than that results from Bahir Dar (9.5%) [31], South Ethiopia (8.3%) [33], and Botswana, Gaborone (21%) [30]. This might be due to variations in personal hygiene and environmental sanitation, and food handling practices of the community and due to climatic differences which affect the viability of infective pathogens.

In our study, children within the age range of 1–3 years were more susceptible to diarrhea caused by *Salmonella* (12.8%) and *Shigella* (7%) isolates. This finding was in agreement with several studies [6, 9, 33, 34]. Children at this age are naturally taking contaminated soils, food and water into their mouth and may acquire disease causing microbes including pathogenic *Salmonella* and *Shigella* spp. from the environment easily. Children from rural areas were more susceptible to diarrhea due to *Salmonella* and *Shigella* infection than children from urban areas, a result in conformity with study of Vargas et al. [35]. The reason might be because less awareness on personal hygiene and environmental sanitation and a reduced provision of health facilitates in rural areas compared to urban areas.

Our study revealed that study subjects who used water from unimproved sources, and had no access to latrine had higher odd of prevalence of *Salmonella* and *Shigella* isolates than their counterparts although the difference was significant for the former factor (Tables 2 and 3). It was indicated that the consumption of contaminated food and/ or water is responsible for diarrheal diseases caused by *Salmonella* and *Shigella* isolates [3, 4]. Children whose parents did not wash their hands before serving food to them and after toilet had higher odd of prevalence of *Salmonella* and *Shigella* spp. in concordance with several studies [8, 9, 15–17]. This was a result of fecal-oral transmission of these bacteria from the parents to their children during feeding and/or handling [3, 4]. Children whose parents didn’t possess waste disposal facility and reared domestic animals had higher odd of prevalence of *Salmonella* and *Shigella* isolates although the difference was not significant as compared to their counter parts (Tables 2 and 3). This was in concordance with studies in Arba Minch [9], in Thailand [15] and in Guinea-Bissau [36]. It is revealed that unhygienic living circumstances and close relations between humans and animals may substantially contribute to the occurrence of salmonellosis and shigellosis [11, 37].

The antimicrobial resistance of enteric pathogens such as *Salmonella* and *Shigella* have been increased all over the world as a result of indiscriminate use of antimicrobial agents [18, 32, 34, 35, 38]. High level of resistance of *Salmonella* isolates to amoxicillin (100%) revealed in this study was comparable with studies in different areas of Ethiopia (100%) [6, 9, 21]. The resistance of *Salmonella* isolates against chloramphenicol (24.1%) was comparable with study in Addis Ababa (21.7%) [8], but lower than earlier studies in Harar (62.3%) [21] and in Addis Ababa (83.7%) [18]. The current use of this drug is limited in RGH and GRH. The rate of resistance of *Salmonella* isolates to tetracycline in our study (79.3%) was higher than in Harar (71.4%) [21], in Hawassa town (0%) [7] and in Mozambique 15% [28]. It was revealed that antibiotic resistance of *Salmonella* isolates from diarrheic children in Southeastern Africa was conferred by *tem-*like β-lactamases for ampicillin, *floR* genes and CAT activity for chloramphenicol, *tetA* genes for tetracycline, and *dfrA1* genes for trimethoprim-sulfamethoxazole/ co-trimoxazole [28]. The susceptibility of *Salmonella* isolates in our study to ciprofloxacin and ceftriaxone (100%) was higher and consistent with some studies in Ethiopia and elsewhere [6, 8, 28], However, ciprofloxacin resistant (6.67%) *Salmonella* isolates was reported from Nekemte Hospital [20]. It was reported earlier that ciprofloxacin was effective and well tolerated for treatment of typhoid fever in children [39, 40]. In addition ceftraxione resistant isolates were reported from Hawassa town (75%) [7] and Nakemete Hospital (3.33%) [20]. Ceftriaxone is safe to use, including in children, is slowly bactericidal against *Salmonella* serovar Typhi in vitro, and is able to penetrate and kill intracellular bacteria [41].

Our study showed that *Shigella* isolates were 100% resistant to three antibiotics (amoxicillin, chloramphenicol, and tetracycline). The resistance to amoxicillin in our study (100%) was very high and in conformity with previous studies in Ethiopia [6, 7]. The resistance of *Shigella* isolates to tetracycline in our study (100%) was also higher than earlier studies in Addis Ababa (97.3%) [18], in Bahir Dar (93.8%) [19], in Gonder (89.7%) [17] and in Mozambique 66% [28]. Similarly, the resistance of *Shigella* isolates to chloramphenicol (100%) was higher than studies in Arba Minch (62.5%) [9], and in Gonder (67.8%) [14]. The resistance of *Shigella* isolates to many drugs is due to the widespread use of antibiotics in medicine, veterinary medicine, and agriculture [3, 42]. *Shigella* isolates produce R plasmids that code for several resistance genes and can confer multiple antibiotic resistance [3]. Similarly, analyses of antibiotics resistance genes of *Shigella* isolates causing diarrhea in children under age of 5 years in South eastern Africa revealed the presence of *oxa-1*-like β-lactamases for ampicillin, *dfrA1* genes for trimethoprim-sulfamethoxazole/co-trimoxazole, *tetB* genes for tetracycline and Chl acetyltransferase (CAT) activity for chloramphenicol [28]. All *Shigella* isolates in our study exhibited 100% susceptibility to ciprofloxacin and ceftriaxone, a result consistent with studies in Ethiopia [6, 19, 20] and in Mozambique [28]. A relatively higher rate of resistance of *Shigella* isolates to ciprofloxacin than our study was reported from Mekelle Hospital (6.7%) [16] and Gonder Hospital (9.2%) [22], and to ceftraxione was reported from Hawassa town (54.5%) [7] and Addis Ababa (4.3%) [8].

In our study, *Salmonella* isolates (79.3%) and *Shigella* isolates (100%) exhibited multidrug resistant pattern (Table 5). The multidrug resistant pattern of *Salmonella* isolates in our study were lesser than reported from Haramaya, Eastern Ethiopia (85.7%) [43], but higher than Mozambique (23%) [28]. However, the *Shigella* isolates in our study exhibited higher multidrug resistant pattern than those isolated from Haramaya (85.7%) [43], from Mozambique (65%) [28], from Mekele Hospital (80%) [16] and from Gonder University Teaching Hospital (79%) [13]. There are also several reports on multiple antimicrobial resistance among strains of *Shigellia* and *Salmonella* species in Ethiopia [5–9, 31, 33, 34].

The treatment of diarrheal diseases caused by *Salmonella* and *Shigellla* species should consider the age restrictions associated with usage of certain antimicrobials in children. Empiric therapy may be started with oral co-trimoxazole or metronidazole, but in severe cases parenteral treatment with ceftriaxone or fluoroquinolones (ciprofloxacin) might be considered [39–41, 44]. But, the use of fluoroquinolones and tetracyclines in the empirical treatment of diarrhea in small children is constrained by its adverse effects on musculoskeletal events and its adverse effect on the teeth younger than 8 years of age, respectively [45]. Third generation cephalosporins such as ceftraxione is the drug of choice for treatment of severe acute diarrheal caused by pathogens such as *Salmonella* and *Shigella* spp. and they have fewer adverse effects on children and effective against fluoroquinolone resistant strains of these bacterial species [45].

## Conclusions

The overall prevalence of *Salmonella* and *Shigella* isolates was 6.9 and 4.3%, respectively. Children in the ages of 1 to 3 years had relatively higher isolation rate of both *Salmonella* and *Shigella* isolates compared to other ages. Drinking from an unimproved water sources, absence of latrine, absence of hand washing habit before meal and after toilet by parents, and unimmunized children resulted in higher odd of prevalence of *Salmonella* and *Shigella* infection in children. The *Salmonella* and *Shigella* isolates displayed high rate of resistance to commonly used drugs such as ampicillin,tetracycline, chloramphenicol and doxycycline. This indicates that these drugs have a reduced efficacy in the treatment of diarrhea caused by *Salmonella* and *Shigella* isolates. But, all *Salmonella* and *Shigella* isolates were highly susceptible to ciprofloxacin and ceftriaxone drugs of choice recommended for diarrheal diseases caused by these pathogens in the two hospitals. Multiple antimicrobial resistances were high among *Salmonella* and *Shigella* isolates. The limitations of this study were failure of identification of Serogroup of *Salmonella* and *Shigella* species as a results of financial constraints and lack of laboratory facilities in the study areas. Periodic awareness on personal hygiene and environmental sanitation, frequent microbiological analyses of food and water are suggested to reduce diarrhea caused by *Salmonella* and *Shigella* spp*.* The choice of the drugs for the treatment of diarrhea caused by *Salmonella/Shigella* isolates should be supported by an in vitro susceptibility studies of individual drugs.

## Supplementary information

**Additional file 1.** Consent form and socio-demographic data and clinical morbidities of children aged below five years with diarrhea attending Robe General Hospital and Goba Referral Hospital, South East Ethiopia.

## Data Availability

The original data for this study is available from the corresponding author.

## Abbreviations

(C): Chloramphenicol
(DO): Doxycycline
AML: Amoxicillin
AOR: Adjusted Odds Ratio
BRGH: Bale Robe General Hospital (BRGH)
CI: Confidence Interval
CIP: Ciprofloxacin
CLSI: Clinical Laboratory Standards Institute
COR: Crude Odds Ratio
CTR: Ceftriaxone
GRH: Goba Referral Hospital
HE: Hektoen Enteric Agar
MDR: Multi-Drug Resistance
SPSS: Statistical Package for Social Sciences
SS Agar: Salmonella-Shigella Agar
TE: Tetracycline
UK: United Kingdom
VIF: Variance Inflation Factor
WHO: World Health Organization
