# Supplementary material for: Prevalence and antimicrobial susceptibility patterns of Salmonella and Shigella isolates among children aged below five years with diarrhea attending Robe General Hospital and Goba Referral Hospital, South East Ethiopia
Source: Trop Dis Travel Med Vaccines. 2019 Nov 20;5:19. doi: 10.1186/s40794-019-0096-6 (PMC6864936; doi:10.1186/s40794-019-0096-6)
Supplement: Supplementary file 1 — Additional file 1. Consent form and socio-demographic data and clinical morbidities of children aged below five years with diarrhea attending Robe General Hospital and Goba Referral Hospital, South East Ethiopia. [file 40794_2019_96_MOESM1_ESM.docx]

Additional File 1. Consent form and socio-demographic data and clinical morbidities of children aged below five years with diarrhea attending Robe General Hospital and Goba Referral Hospital, South East Ethiopia

**
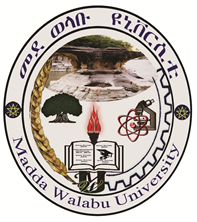
**

**Madda Walabu University**

**College of Natural and Computational Science**

**Department of Biology**

**Semi-structured questionnaire (to be filled by experienced physician/Nurse)**

**Dear respondents (children’s parents or guardians)**

The primary purpose of this questionnaire is to gather primary information on the Socio-demographic and clinical morbidities associated among children aged below five years with diarrhea attending Robe General Hospital and Goba Referral Hospital, South East Ethiopian to the research work needed to the partial fulfillment of Degree of Masters of Science in Applied Biology BY Mr. Mengistu Girma. The success of this research depends on your genuine response and the result of this study will help to provide important recommendations on factors associated prevalence of diarrhea caused by *Salmonella* and *Shigella* isolates. The researcher promises that the entire data gathered will only be used for academic purpose and be confidential.

NB. Before participation in this study, you are requested give us an oral and written consent to participate in the study. Information of the study subjects and that of the parents or guardians of the study subjects were kept confidential. You are eligible to fill this questionnaire if your child had not taken any antibiotics two weeks before the study and complete demographic and clinical morbidities that was related with diarrhea could be provided. You are asked to bring stool specimen of your child to bring freshly passed stool and rectal swab of the study subjects in a sterile stool cup using clean applicator stick provided to you.

**Dear respondents (children’s parents or guardians)**

- Are you willing to participate in the study? Yes/ No
- If your response is Yes, confirm your approval by your name and Signature

Name of children’s parents/ guardian _______________ Signature_______ Date________

**General direction**

- You do not need to write your name or child name.
- Please put an ‘x” mark in the boxes provided against the alternative you choose.
- Please write response briefly in the space provided for questions requiring short answer.

**Thank you in advance!!**

**Dear respondents (children’s parents or guardians)**

**Direction:** The following are some items about your background information of child. You are required to indicate your response by filling the blank space or by encircling the letter of your appropriate answer for each question.

1. **Data on socio-demographic characteristics**
2. Age of the children:______________
3. Sex of the children:______________
4. Your relation with the child: A. Mother B. Father C. Sister D. Brother e. Other

4. Residence of your child A. Urban B. Rural

**B. Data on associated and risk factors for diarrhea**

1. Does you child have diarrhea?

A. Yes B. No

1. What is the main source of your water supply?

A. Improved water source (pipe water or water drunk after physical or chemical treatment)

B. Unimproved water source (river, spring or well water etc drunk without any treatment)

1. Do you have latrine for you family member?
2. Yes B. No.
3. Do you have a waste disposal facilities in your household?

A.Yes B. No.

1. What do you feed your child?
2. Milk B. Exclusively breast feeding C. Both intermittently
3. If you feed milk, how do you use fed your child?
4. Feed after boiling B. Feed without boiling
5. Do you have a habit of washing hands before feeding child?
6. Yes B. No

8. Do you have a habit of washing hands before feeding child?

1. Yes B. No
2. Are you living together in the same house with cattle, calves, goats, and sheep or other domestic animals?

A.Yes B. No

1. Have you child immunized for any types of diseases?
2. Yes B. No
